# Supplementary material for: Are carbon emissions trading and green financial instruments synergistic? -Comprehensive quantitative research based on content analysis
Source: PLoS One. 2024 Mar 7;19(3):e0298601. doi: 10.1371/journal.pone.0298601 (PMC10919601; doi:10.1371/journal.pone.0298601)
Supplement: S1 Data — (ZIP) [file pone.0298601.s001.zip › Data/地级市Z_F工作报告.zip!/║╙─╧/╓ú╓▌╩╨/└┤╫╘╣1⁄2╓┌║┼í╛┬φ┐╦╩2╛▌═°í┐_2011.docx]

现在，我代表市人民政府，向大会作工作报告，请予审议，并请各位政协委员和列席人员提出意见。

　　一、2010年及“十一五”工作回顾

　　2010年，面对复杂形势和严峻挑战，在省委、省政府和市委的正确领导下，全市上下深入贯彻落实科学发展观，继续大力实施跨越式发展战略，团结拼搏，克难攻坚，较好地完成了市十三届人大二次会议确定的目标任务，经济社会继续保持跨越式发展的良好态势。初步统计，全市实现生产总值4000亿元，增长13%。财政总收入完成643亿元，增长23.2%，其中，一般预算收入完成386.8亿元，增长28.1%。规模以上工业完成增加值1600亿元，增长18%；实现利税1009亿元，增长27.1%。全社会固定资产投资完成2757亿元，增长20.8%。实现社会消费品零售总额1678亿元，增长19%。市属及以下企业出口总额达到33.1亿美元，增长62.8%。城镇居民人均可支配收入达到18897元，增长10.4%；农民人均纯收入达到9225元，增长13.6%。全市万元生产总值能耗下降3.6%，化学需氧量和二氧化硫排放量控制在省定目标以内。

　　（一）结构调整和产业升级迈出新步伐。在保持经济平稳较快发展的同时，更加注重经济结构调整和产业转型升级，着力增强产业竞争力。工业结构进一步优化。全面实施产业调整振兴规划，加快新型工业化进程。新开工转型升级项目86个，竣工98个，完成投资457.5亿元。富士康、宇通、海马、东风日产、郑州日产、中国龙工、格力电器、旭飞光电、保绿能源等企业的一批重大项目开工建设，为全市工业经济结构调整奠定了基矗扶优扶强成效明显，年销售收入超百亿元企业达到7家，其中，郑煤、宇通、中烟等3家企业销售收入均超过200亿元。现代服务业快速发展。我市被确定为国家服务业综合改革试点城市。现代化商贸城总体规划、商业网点规划及城区商业网点控规和郑州国际物流园区、航空港物流园区等功能区控规编制进展顺利，新加坡国际物流园被正式确定为国家层面战略合作项目，中南邮政物流集散中心开工建设。38个重点商贸物流项目实现投资78.1亿元，完成年度投资计划的127.4%。金融中心建设稳步推进，全市银行类金融机构达到24家；郑州商品交易所期货交易规模达到61.8万亿元，增长223.4%；地方金融机构发展步伐加快，郑州银行成立多家县域支行；郑煤机等4家企业成功上市，全市上市公司达到30家。成功举办了第二届中国绿化博览会、第八届中国国际农产品交易会、台湾产品月、中国国际汽车后市场博览会等100余场展会，展会面积达到132万平方米。旅游业快速发展，全年旅游总收入达到510亿元，增长16.4%。现代农业稳步推进。粮食生产再获丰收，连续8年实现增产。加快农业发展方式转变，编制实施了都市型现代农业发展规划以及蔬菜、花卉、水产等专项规划。新建3个现代农业示范园区，新增45家市级以上农业龙头企业，新增428个农民专业合作社。推进农业生产由小规模分散经营向规模化经营转变，全市土地流转面积达到36万亩。非公有制经济规模不断壮大。拓宽中小企业融资渠道，完善中小企业信用担保体系，加强企业服务，支持非公有制企业发展。全市非公有制经济完成增加值2400亿元，增长13.4%，占全市生产总值的60%。对外开放和招商引资成效明显。郑州新郑综合保税区获国务院批准，成为中部地区第一个综合保税区，河南保税物流中心（B型）园区一期项目通过国家验收，出口加工区B区加快建设，综合保税区、保税物流中心和出口加工区成为我市对外开放的三大平台。坚持“大招商、招大商”，以国内外500强企业为重点，瞄准央企和港澳台大项目，积极承接产业转移。实际利用外商直接投资19亿美元，增长17%；引进市外境内资金721亿元，增长18.4%。成功引进富士康项目，实现了承接产业转移和招商引资的重大突破，在洽谈、签约、厂房建设以及综合保税区申建等方面创造了“郑州速度”。

　　（二）经济发展方式转变取得新进展。把加快经济发展方式转变作为贯彻落实科学发展观的核心和关键环节，不断提高经济发展质量和效益，增强可持续发展能力。产业实现集聚发展。科学编制并实施产业集聚区各项规划，在基础设施、融资担保、财政激励等方面加大扶持力度，进一步理顺管理体制，产业集聚区承载功能不断完善，已成为新的经济支撑点。38个产业集聚区基础设施投入达到172亿元，增长30.1%；规模以上企业销售收入完成3075.7亿元，增长31.2%。全市年销售收入超百亿元集聚区达到18个。消费需求进一步扩大。积极实施促进消费的政策措施，加大“万村千乡”市场工程和家电下乡、汽车和家电以旧换新、特色商业街建设等工作推进力度。促进房地产业健康发展，房地产销售面积达到1558.7万平方米，增长29.8%。自主创新能力得到加强。大力实施科技自主创新工程，68个重点项目完成投资46.4亿元，占年度计划的127%。实施企业自主研发项目145项，郑煤机液压支架电液控制系统等14个项目达到国际先进水平。新批建设国家级重点实验室1家，新认定省级工程技术研究中心21家。我市被确定为国家创新型试点城市，高新区被确定为第一批国家创新型园区。知识产权专利授权量增长49.2%，荣获国家知识产权示范市称号。节能减排和生态建设力度进一步加大。大力淘汰落后产能，兼并重组煤炭企业133家，关闭、取缔污染严重的企业396家。十七里河、熊儿河、金水河等主要河道实现截污排放，投资14.4亿元的生态水系工程已具备通水条件，河岸绿化工作全面展开。加强水资源保护工作，我市被评为全国节水型社会建设示范城市。新造林19万亩，建成12个林业生态乡镇、100个林业生态村。郑州绿博园建成开园。中心城区新增绿地635万平方米，空气质量优良天数达到318天，饮用水源地水质达标率保持100%。

　　（三）城乡面貌发生新变化。全面实施城市总体规划，加快编制郑州新区规划，完善城市功能，提升城市品位，城镇化水平进一步提高。郑州新区规划建设快速推进。郑汴新区总体规划经省人大常委会审议通过，综合交通、城镇体系、产业发展等30项规划的编制工作顺利完成，形成较为完善的规划体系。推进龙湖地区、高校和职教园区、航空港区、汽车及装备制造产业园区等重点区域的开发建设，在重大项目布局、基础设施配套、土地征用等方面取得了新的突破。加快新区基础设施配套建设，各个组团基础支撑与保障能力不断增强。中心城区建设和管理水平进一步提高。强力推进“畅通郑州”工程，不断完善城市交通基础设施，有效改善了城市交通状况。轨道交通1号线一期工程除东明路站外的所有站点全部开工，2号线开工建设。科学大道—西三环立交、东风路—文化路下穿隧道、中州大道北段综合整治、解放路立交等工程相继竣工通车。京沙快速通道全线开工，部分路段投入使用。客运北站开工建设，客运南站建成投入使用。列入计划的20条断头路全部打通，又开工26条。完成雨污水改造工程33项，完成背街小巷道路改造106条。新购公交车661辆，公交线路达到225条。王新庄、五龙口、马头岗等污水处理厂实现不间断达标运行。新建垃圾中转站26座、改造28座，敷设供水管道47公里，新建、改建市区燃气管网121.6公里，新建供热管网35公里。城中村、旧城和棚户区改造步伐不断加快。开展占道经营、户外广告、垃圾运输车和环境卫生整治，加快数字城市建设，城市精细化管理水平进一步提高。交通枢纽地位不断强化。郑西客专投入运营，石武客专、郑民高速、郑州综合交通枢纽公路客运站等工程顺利推进，郑州至焦作、郑州至开封城际铁路相继开工建设，黄河公铁两用桥公路桥和花园口互通式立交新建工程主线建成通车。郑州机场建设步伐加快，旅客吞吐量突破870万人次，年货邮吞吐量达到8.58万吨。农村生产生活条件得到改善。改造中低产田13万亩，新建、改建农村公路578公里，解决了农村51万人的饮水安全问题。完成29个贫困村的整村推进和1992户7295人的扶贫搬迁任务，1.3万人实现脱贫。完成127个新农村建设项目。深入开展文化、科技、卫生“三下乡”活动，建成500个农村文化大院、299个农家书屋。

　　（四）民生保障和社会建设实现新突破。始终坚持经济发展与民生改善相统一，经济发展与社会建设相协调，让人民群众共享发展成果。向社会承诺为群众办好的“十大实事”，件件得到落实，群众关注的一些热点、难点问题得到有效解决。就业工作扎实推进。落实扶持就业的各项优惠政策，新增城镇就业13.4万人，农村劳动力转移就业11.2万人。以创业带动就业，发放小额担保贷款8亿元。开展系列就业服务活动，促进高校毕业生就业。零就业家庭动态为零。城镇登记失业率控制在3%以内。社会保障不断完善。新型农村合作医疗人均筹资标准提高到150元，参合率达到98%。城乡居民养老保险范围进一步扩大，企业退休人员基本养老金人均月增加148元，城市和农村低保人均标准分别提高15元、10元。加强保障性住房建设，经济适用房新开工166万平方米，竣工112万平方米；开工建设廉租住房15.4万平方米，竣工5.7万平方米。加强物价监测和管理，严厉打击价格违法行为，发放物价补贴，保障了低收入群体及困难群众的基本生活。社会事业加快发展。制定实施教育、人才改革发展规划。市区新建、改扩建15所中小学校全部开工，顺利实施校舍安全工程，实施中小学校现代远程教育“班班通”工程。在全省率先实现郑州户籍中职生免费教育。郑州师专升格为郑州师范学院，郑州幼师新校区开工建设。基本公共卫生服务不断加强，基层医疗卫生条件得到改善。新建27个片医负责制单位，全市588个片医小组服务居民达280万人。郑州图书馆新馆（市民文化中心）完成主体工程建设，新建、改建100个社区文化活动中心，公共文化服务体系更加完善。登封“天地之中”历史建筑群成功列入世界遗产名录。成功举办了庚寅年黄帝故里拜祖大典和第八届中国郑州国际少林武术节。竞技体育和群众体育取得新成绩，我市培养和输送的运动员在第十六届亚运会上共获得7枚金牌。低生育水平持续稳定，人口自然增长率为5.2‰，我市被确定为全国人口和计划生育综合改革示范市、全国流动人口均等化服务综合改革试点城市。社会大局和谐稳定。严格落实安全生产责任制，安全生产形势总体平稳。加强食品药品安全管理，产品质量和食品安全保障水平继续提高。信访工作机制不断创新，应急管理体制不断完善。推进平安郑州建设，完善社会治安综合防控体系，深入开展“四严一创”活动，命案破案率达97.12%，群众安全感和满意度大幅提升，我市成为全国最具安全感的城市之一。

　　（五）民主法制和精神文明建设得到新提升。自觉接受人民代表大会及其常委会的法律监督和工作监督，接受人民政协的民主监督，认真听取民主党派、工商联、无党派人士和各人民团体的意见。全年办复市人大代表议案、建议和政协委员提案1206件。坚持依法治市，社会法治化管理水平不断提高，提请通过地方性法规2件，制定政府规章16件。廉政建设和反腐败斗争取得新成效。坚持“三具两基一抓手”，深入推进“两转两提”，认真开展“创先争优”活动，团结拼搏、干事创业的氛围更加浓厚。加强精神文明建设，公民道德水平和文明程度不断提高。

　　大力开展国防教育，积极做好“双拥”工作。驻郑解放军和武警部队在支持地方经济建设、维护社会稳定方面作出了重要贡献。外事、侨务、对台工作不断加强，民族、宗教、统计、史志、文物、地质、气象、地震、人防、社科研究等工作取得新成绩。高标准完成省下达的南水北调征地拆迁和移民安置任务。第六次人口普查工作进展顺利。圆满完成四川江油四镇灾后援建工作。

各位代表！

　　2010年各项工作目标的顺利实现，标志着我市“十一五”主要任务圆满完成。过去的五年，面对各种复杂形势和困难局面，特别是面对国际金融危机的严重冲击，我们发扬顽强拼搏的精神，凝心聚力，开拓创新，办成了一批具有深远意义的大事，办好了一批深受群众欢迎的实事，办妥了一批前所未有的难事，谱写了我市全面建设小康社会的崭新篇章。

　　——过去的五年，是全市经济社会不断实现新跨越、综合实力显著增强的五年。全市生产总值由不足2000亿元到突破4000亿元，年均增长13.7%。财政一般预算收入是“十五”末的2.8倍，全部工业增加值由不足800亿元到2000亿元，金融机构各项存款余额由3000亿元到8000亿元。

　　——过去的五年，是经济结构加速调整、发展方式加快转变的五年。以构建“一个载体、三个体系”为重点，深入推进经济结构调整和发展方式转变。产业结构不断优化，二、三产业比重由95.6%提高到96.9%。自主创新能力不断增强，全市专利申请量和专利授权量分别达到8203件和5677件，比“十五”末分别增长233%和429%。单位生产总值能耗累计下降20.4%，“十一五”节能减排目标顺利完成。

　　——过去的五年，是城乡面貌日新月异、城市形象大幅提升的五年。建成区面积由262平方公里扩大到316平方公里，城镇化率提高7.2个百分点。开工建设了一大批道路桥梁工程，水电气暖保障能力明显提升，污水处理和垃圾处理设施不断完善。城市生态水系基本形成，市区河段初步实现“水通、水清”目标，提前4年完成森林生态城100万亩林木栽植任务。郑东新区顺利实现“三年出形象、五年成规模”的目标，成为展示郑州乃至河南的名片。郑州新区建设顺利启动。我市先后荣获国家卫生城、国家园林城、全国绿化模范城称号。新建、改建农村公路累计达到5989公里，解决159万人饮水安全问题，投入130亿元完成了332个新农村建设项目，农村面貌得到明显改善。

　　——过去的五年，是各项改革深入推进、对外开放取得重大成果的五年。国企改革迈出新步伐，210户国有企业成功实现改制重组，一批困难企业焕发出新活力。创新投融资模式，组建投融资公司9家，累计融资353.5亿元。坚持“大招商、招大商”，累计引进外资项目577个，富士康、日产、杜邦、汇丰等19家世界500强企业在郑投资，国开投、东风、海马等95家国内500强企业在郑设立机构和投资，中石油、中石化、中粮、中铁等34家央企在郑投资，实际利用外商直接投资由3.4亿美元到19亿美元。鼓励企业对外投资，境外投资涉及42个国家和地区。

　　——过去的五年，是民生保障进一步加强、人民群众得到实惠最多的五年。公共财政用于教育、社会保障、就业、医疗卫生等民生领域的支出累计达500亿元。“十大实事”累计投入135亿元，一大批群众关心的热点问题得到有效解决。城镇累计新增就业63.5万人，农村劳动力转移就业62万人。新建、改扩建中小学校70所。在全国率先推行了市属国有破产和困难企业退休人员大病统筹医疗保险，在全国省会城市率先建立了城乡居民养老保险制度，民生保障体系初步形成。建成经济适用房454万平方米、廉租住房13万平方米，累计有3万余户低收入住房困难家庭享受到了廉租住房政策。安全生产水平提高，治安防控能力增强，社会大局和谐稳定。

　　回顾五年来的奋斗历程，特别是应对国际金融危机的实践，进一步深化了我们对贯彻落实科学发展观的认识，提高了应对复杂局面的能力，坚定了跨越式发展的信心和决心。我们有以下体会：一是始终坚持以科学发展观为统领，不断提高可持续发展能力。坚持把发展作为解决一切问题的关键，创新发展思路，突破发展瓶颈，转变发展方式，突出发展效益，千方百计保持经济又好又快发展。二是始终坚持实施跨越式发展战略，不断增强干事创业的凝聚力。大力实施跨越式发展三年行动计划，以重点工程为抓手，以重大项目为载体，凝聚全市力量，推动经济社会发展实现新跨越。三是始终坚持改革开放不动摇，不断强化发展驱动力。把改革开放作为解决深层次矛盾的根本对策，以改革创新破解发展难题、强化发展保障，以扩大开放加快发展步伐、增创发展优势。四是始终坚持自主创新，不断夯实科技支撑力。全面提高自主创新能力，实现资源资本推动向科技推动转变、粗放管理向科学管理转变、经营产品向经营品牌转变。促进产学研紧密结合，集中力量攻克带动性强、关联度大、作用突出的关键技术，用科技创新加速推进经济社会发展。五是始终坚持改善民生这个根本，不断提升公共服务能力。把保障和改善民生作为发展的根本出发点和落脚点，逐年提高财政用于民生的投入，每年坚持为人民群众办理“十大实事”，以改善民生凝聚民心，以凝聚民心汇聚民力，以汇聚民力共谋发展。

各位代表！

　　五年取得的成绩来之不易，积累的经验弥足珍贵，创造的精神财富影响深远。“十一五”期间取得的成就，是全面贯彻落实党和国家路线方针政策的结果，是省委、省政府和市委正确领导的结果，是市人大、市政协监督支持的结果，是全市人民共同努力的结果。在此，我代表市人民政府，向工作在各条战线上的工人、农民、知识分子和干部，向驻郑人民解放军、武警官兵、公安政法干警，向各民主党派、工商联、人民团体和各界人士，向关心和支持郑州发展的港澳台同胞、海外侨胞和国际友人，表示衷心的感谢和崇高的敬意！

　　在回顾成绩的同时，我们也清醒地看到经济社会发展中的一些困难和问题。一是结构性矛盾仍然比较突出，高耗能、资源型工业比重较大，现代服务业发展不够充分，加快发展方式转变任务艰巨。二是瓶颈制约因素较多，资金、土地和环境等要素约束进一步加剧，一些重要领域的体制机制障碍仍然存在。三是创新能力还不强，高科技企业和高端科技人才相对不足，科技创新和科技成果转化能力有待提高。四是公共服务能力有待进一步提升，上学难、看病难等民生问题依然存在，城市交通问题尚未得到有效解决，水电气暖等基础设施需要进一步完善。五是一些政府部门职能转变还不到位，自身建设有待加强。这些问题，都需要在今后的工作中高度重视并认真加以解决。

二、“十二五”发展的指导思想和目标任务

　　“十二五”时期是贯彻落实科学发展观、加快经济发展方式转变、全面建设小康社会的关键时期，是建设郑州都市区、打造中原经济区核心增长极、加快建设国家区域性中心城市的攻坚时期，也是必须紧紧抓住并可以大有作为的重要战略机遇期。面对新形势、新要求，我们必须以世界眼光和战略思维，发挥优势，抢抓机遇，乘势而上，继续保持经济社会跨越式发展的良好态势。

　　“十二五”时期我市经济社会发展的指导思想是：高举中国特色社会主义伟大旗帜，以邓小平理论和“三个代表”重要思想为指导，深入贯彻落实科学发展观，按照“四个重在”的实践要领，紧紧抓住中原经济区建设重大机遇，以科学发展为主题，以加快经济发展方式转变为主线，以建设国家区域性中心城市为总目标，以富民强市为中心任务，以加快建设郑州都市区、持续推进跨越式发展、形成中原经济区核心增长极为总体思路，着力扩大需求，着力优化结构，着力改革开放，着力自主创新，着力环境保障，着力改善民生，充分发挥龙头作用、重心作用和示范带动作用，促进经济又好又快发展，促进社会和谐稳定和全面进步，为全面建成更高水平的小康社会打下坚实基础。

　　主要目标是：生产总值年均增长13%，财政总收入年均增长13%，财政一般预算收入年均增长13%，规模以上工业增加值年均增长16%，全社会固定资产投资年均增长20%，社会消费品零售总额年均增长15%，进出口总额年均增长25%，实际利用外商直接投资年均增长20%，城镇居民人均可支配收入年均增长10%，农民人均纯收入年均增长10%，新增城镇就业65万人，转移农村劳动力50万人，万元生产总值能耗完成省下达目标。

　　实现上述目标任务，必须突出以下几个方面：

　　一是大力推进郑州都市区建设。按照组团发展的理念，以推进交通基础设施和生态环境建设为重点，加速形成以中心城区和外围组团为核心、中等城市为支撑、重点镇为节点、小城镇为拱卫的现代大都市，合理调整城市空间布局和功能分区，努力形成组合有序、优势互补、整体协调的发展新格局。

　　二是切实转变经济发展方式。坚持把经济结构战略性调整作为主攻方向，坚持把科技进步和自主创新作为重要支撑，坚持把建设资源节约型、环境友好型社会作为重要着力点，加快推进创新型城市和低碳生态城市建设，切实做到在发展中促转变、在转变中谋发展。

　　三是加快富民强市步伐。坚持富民优先、强市为要，在不断增强我市综合实力的基础上，千方百计增加居民收入。大力实施“畅通郑州”工程、“菜篮子”工程、安居工程和教育、医疗、社会保障等民生工程，提高公共服务供给能力，推进公共服务均等化。

　　四是继续深化改革开放。把改革创新作为破解发展瓶颈和难题的根本途径，坚定不移地推进重点领域和关键环节的改革，建立健全有利于转变经济发展方式、促进科学发展的体制机制，最大限度地解放和发展生产力。把对外开放作为借势跨越、加快发展的根本举措，积极实施开放带动主战略，加大招商引资力度，承接产业转移，构建全方位、多层次、宽领域的开放格局。

　　五是进一步优化发展环境。继续实行联审联批、限时办结、审批代办、首席服务官和重大项目领导分包等制度，完善绩效考评和奖惩制度，全面提高行政服务效能，切实营造公平正义的法制环境、宽松优良的政策环境、廉洁高效的政务环境、诚实守信的信用环境，努力打造最佳投资环境城市、最佳人居环境城市、最佳创业环境城市和最佳旅游环境城市。

　　《郑州市国民经济和社会发展第十二个五年规划纲要（草案）》及说明已印发各位代表，请予审议。

三、2011年工作安排

　　2011年是实施“十二五”规划的开局之年，也是郑州都市区建设的起步之年。新的一年，世界经济将继续缓慢复苏，我国经济发展持续向好的趋势没有改变，我省“一个载体、三个体系”建设扎实推进，中原经济区建设正式启动。我市区位、交通、资源和产业优势进一步显现，一批事关长远发展的重大项目取得突破性进展，发展动力和活力明显增强，这些都为我们实现科学发展、跨越发展提供了难得机遇。同时，我们也认识到，世界经济复苏的过程中不稳定不确定因素仍然较多，国家宏观调控政策进入转换期，特别是在土地供给、项目审批、节能减排等方面将提出新的要求，经济发展将面临偏紧的宏观环境和要素供给形势。我市面临的区域竞争压力不断加大，存在的矛盾和问题依然较多。因此，我们一定要保持清醒，增强忧患意识，抓住机遇，迎接挑战，顺势而为，扎实工作。坚持以郑州都市区建设为载体，以有效承接产业转移为关键，以项目建设为抓手，把经济结构战略性调整作为转变发展方式的主攻方向，把改造提升传统产业作为调整优化结构的首要任务，把搭建对外开放主平台作为以增量调结构促转型的有效载体，把强化综合交通枢纽地位作为提高区域竞争力打造竞争新优势的重要举措，努力实现“十二五”良好开局，充分发挥在中原经济区建设中的核心和示范带动作用。

　　今年经济社会发展主要预期目标是：生产总值增长13%，财政一般预算收入增长15%，全社会固定资产投资增长23%，社会消费品零售总额增长17%，进出口总额增长25%，实际利用外商直接投资增长22%，居民消费价格涨幅控制在4%左右，城镇居民人均可支配收入增长10%，农民人均纯收入增长10%，新增城镇就业13万人，城镇登记失业率控制在4%以内，人口自然增长率控制在6.5‰以内，节能减排完成省下达目标。

　　围绕上述目标，今年将着重抓好以下几个方面工作：

　　（一）加快郑州都市区建设，打造中原经济区核心增长极。以现代化大都市的战略思维和发展理念，超前谋划并扎实推进郑州都市区建设，主动融入并服务于中原经济区建设。做好规划编制工作。高标准做好郑州都市区空间战略规划、建设规划和交通专项规划，制定新增组团的总体规划，编制宜居教育城、宜居健康城等宜居组团规划。拓展都市区发展空间。加快中心城区和郑州新区发展，加大三环至四环以及四环至黄河南岸、郑州新区、西南绕城高速区域内的城市基础设施建设力度，拉大城市框架，实现延伸发展；推动新郑、荥阳、新密、上街、高新技术开发区等组团的开发建设，推进融合发展；加快巩义、登封等外围组团建设，实施向心发展。加快城乡统筹发展。坚持以产兴城、以城带乡，壮大都市区各组团经济规模。按照城市建设标准，着力推进重点镇向小城市方向发展。加快新农村建设，开展新型农村社区建设。稳步推进合村并城、合村并镇、合村并点，实现人口向城镇和农村社区集中、土地向规模经营集中、产业向集聚区集中，加快城乡一体化进程。

　　（二）推进郑州新区发展，构建复合型新城区。进一步完善专项规划、控制性详规和修建性详规，实现控制性详规全覆盖。加快航空港和高速铁路两大综合交通枢纽建设，加快郑汴物流通道、万洪公路等道路工程建设，推进重大产业集聚区周边道路和水电气等基础设施配套建设，加快郑州引黄灌溉龙湖蓄水工程项目建设。加强城市东出入市口的交通整治和环境综合整治，疏通新区交通瓶颈。集中力量抓好航空港区、龙湖地区、国际物流园区、高校和职教园区、综合交通枢纽地区和绿博园周边地区等重点区域的开发建设，以点带面，推动新区整体发展。全力推进富士康郑州科技园建设，确保按建设进度完成任务，同时，积极引进新的合作项目，形成富士康项目的产业集群。加快东风日产、郑州日产、海马、宇通等整车生产基地建设，加快汽车零部件产业园和新能源汽车研发基地建设，为百万辆汽车生产规模如期达产奠定坚实基础。建设好综合保税区、保税物流中心、出口加工区三大海关特殊监管区。依托郑东新区CBD、CBD副中心，大力发展金融服务业、现代物流业、会展业、总部经济和楼宇经济。

　（三）加强经济运行调控调节，保持经济平稳健康发展。积极扩大消费需求，大力优化投资结构，为经济平稳较快发展创造条件。努力扩大消费需求。增加政府支出用于改善和扩大消费的比重，增强居民消费能力。落实商贸城建设总体规划和城市商业网点规划，出台加快现代化商贸城发展的意见。继续实施家电下乡、以旧换新等刺激消费政策，深入推进“万村千乡”市场工程和新网工程。制定蔬菜流通体系建设规划，加快批发市场、农贸市场、社区便利店建设，推进蔬菜店、早餐店进社区。培育文化、教育、养老、医疗保健等消费热点，完善家政服务体系，争取成为国家家政服务体系试点城市。优化投资结构。发挥好政府投资对经济结构调整的引导作用。认真落实国家关于鼓励引导民间投资的政策措施，引导社会资本进入基础设施、公用事业、保障性住房、金融服务等领域。加强银企衔接，扩大直接融资规模。支持郑州银行等金融机构推进金融产品和服务方式创新，争取更多信贷资金投向实体经济。加强经济运行监控监测。加大煤电油气运等生产要素的保障力度，千方百计帮助企业解决手续办理、土地审批、融资等难题。加强房地产市场宏观调控，强化市场监管，保持房地产市场平稳健康发展。综合运用调控手段，全力保障市场供应，努力把价格总水平控制在合理范围。

　　（四）优化产业结构，提升产业核心竞争力。完善产业政策，构建现代产业体系，推动产业结构调整。发展壮大战略支撑产业。重点发展汽车及装备制造、物流商贸、文化创意旅游和电子信息四大战略支撑产业。加快宇通2万辆客车产能提升、海马第三工厂、东风日产年产60万辆轿车、旭飞光电液晶玻璃基板项目二期、恒天重工、新大方工业园、中船重工核电设备研制生产等项目建设；重点支持郑州铁路集装箱中心站、华润万象城、万达广场等大型物流商贸项目建设，推动美国联合包裹服务公司郑州项目、新加坡国际物流园项目尽快落地；推进郑州华强文化科技产业基地、国家动漫产业发展基地（河南基地）、郑州信息创意产业园等文化产业园区建设。推进富士康郑州科技园、中国电科27所郑州电子信息产业园、威科姆国际软件生态园、河南物联网科技产业园等信息产业项目建设。加快信息化和工业化融合步伐，积极争取国家级云计算中心落户郑州，创建国家电子商务示范城市，推进电子信息产业实现新跨越。培育发展战略新兴产业。重点实施新能源、新材料、节能环保、生物医药、物联网等重大科技专项，努力建成一批标志性科技工程。推进海马新能源汽车、中航电动汽车动力总成系统、郑州日产年产5万辆电动汽车研发及产业化等项目建设，推进桑达能源、华晶超硬材料等一批新能源、新材料项目建设，大力支持辅仁药业科技产业园、安图生物等重点生物医药项目，做好“十城万盏”半导体照明和“十城千辆”新能源汽车试点工作。改造提升传统优势产业。围绕铝及铝制品、食品和纺织服装等传统产业，坚持技术创新、升级改造、延伸链条、降本提效。积极发展煤电铝一体化，开发铝加工高端产品；提升食品工业制作技术，扩大生产规模；大力发展高档面料，培育国际知名品牌，提升服装产业层次。推进豫联集团电解槽升级改造、东方铝业精深加工、中粮（郑州）工业园、益海嘉里、双汇食品工业园、三全食品工业园、思念食品新工业园、泰阳纺织有限公司高档纺织面料生产线等项目建设。大力发展现代服务业。重点发展食品冷链、医药、钢铁、粮食、汽车、家电等行业物流，加快建设专业物流园区、国家级区域性分拨中心和配送网络。着力引进国际国内知名金融机构入驻我市，支持地方金融机构和地方融资平台的发展。以郑州商品交易所为龙头做大做强期货经营机构，加快郑州期货定价、信息和交易中心建设。调整优化商贸布局，大力发展城市综合体。推动会展业加快发展，举办好全国药品交易会、中国国际五金博览会、中国国际汽车后市场博览会等展会。加快旅游基础设施建设，完善配套服务功能，加快与港中旅合作项目的推进，再引进一批国内外知名的新型旅游业态项目。加快实施自主创新。围绕国家创新型城市建设，推进重大科技创新工程，实施重点科技攻关计划，加快产学研结合及科技成果转化。支持中铁隧道盾构技术、宇通客车安全控制技术、郑州机械研究所等国家级和省级企业研发中心、工程实验室建设。力争全市高新技术产业总产值突破2500亿元，专利授权量突破6000件，科技进步贡献率达到56%。

（五）狠抓重大项目建设，加大固定资产投资力度。把项目建设作为推进跨越式发展的重要抓手，以项目调结构，以项目促增量。加强综合交通基础设施建设。继续配合国家、省加快石武客专、郑徐客专、郑州机场二期以及机场至许昌、机场至登封、机场至洛阳城际铁路等综合交通体系建设，推进郑州至开封、郑州至焦作、郑州至机场城际铁路项目建设，配合做好郑渝铁路前期工作，配合省加快连霍高速、焦桐高速、郑民高速郑州段等项目建设，开工建设机场至少林寺高速公路。完成国道107郑州段改建等快速路项目，加快推进郑新快速通道、航海路至郑少高速连接线等快速路项目。加快郑州综合交通枢纽公路客运站等项目建设。加快基础产业发展。做好国电荥阳电厂二期2×100万千瓦、大唐巩义2×60万千瓦发电机组等项目的前期工作，推进新密电厂二期2×100万千瓦、河南华辕年产240万吨李粮店煤矿等重大项目建设，确保海马郑州年产15万台节能高效发动机、格力电器郑州产业园一期、友旺手机产业园、郑煤机高端液压支架基地等25个亿元以上项目如期竣工达产，开工建设中国家居CBD郑州产业园、保绿能源二期、河南龙工中原基地、青岛海尔（郑州）物流产业园一期等项目。推进产业集聚区建设。按照企业集中布局、产业集群发展、资源集约利用、功能集合构建和促进人口向城镇转移等“四集一转”的要求，完善产业集聚区推进机制和管理办法，整合产业资源，培育特色产业集群。加快产业集聚区服务平台建设，加大产业集聚区招商引资力度。

　　（六）扎实做好“三农”工作，夯实农业农村发展基础。在工业化、城镇化加快发展的同时，进一步加快现代农业和都市农业发展，推动“三化”协调发展。认真落实强农惠农政策。财政支出重点向农业农村倾斜，确保用于农业农村的总量、增量均有提高。加强农村金融体系建设，引导农村信用社、县域村镇银行等金融机构支持“三农”发展，推进农业政策性保险工作，发展农产品期货市场，保护和调动农民发展农业生产的积极性。加快都市型现代农业发展。加快实施近郊蔬菜、花卉、水产等发展规划。大力实施“菜篮子”工程，新建、改建标准化菜田2万亩，其中，发展设施生产面积8000亩。抓好中牟现代农业示范区起步区和中国红枣综合产业园等农业特色区建设。加大对龙头企业的扶持力度，大力发展农民专业合作社。强化农业科技支撑，健全基层农业技术推广服务体系。加强农业农村基础设施建设。大力开展农田水利基本建设，加快推进病险水库除险加固、中小河流治理、水土保持等项目建设，完成中央投资大型泵站改造和引黄灌区节水配套改造项目。新修、改造农村公路500公里。加大农业综合开发力度，改造中低产田20万亩，治理水土流失面积80平方公里，新增有效灌溉面积3万亩。继续为南水北调工程建设创造良好环境，全面完成第二批移民搬迁安置任务。加大扶贫开发力度，帮助1万人实现脱贫。

　　（七）加强城市建设与管理，提升城市综合承载能力。以郑州都市区规划建设为契机，加快中心城区保护改造步伐，着力提升城市管理水平。完善实施各项规划。认真落实郑州市城市总体规划，做好各层次规划的衔接。尽快完成郑州航空城空间战略规划及城市设计，上半年编制完成市区城乡空间发展引导规划。继续实施“畅通郑州”工程。加快轨道交通建设，力争1号线全线贯通，开工建设1号线二期和4号线部分市政工程，做好5号线前期工作。完善城市快速路系统，升级改造10个出入市口，推动郑开大道与京港澳高速、花园路与连霍高速等互通式立交建设，力争京沙快速通道一期工程建成投入使用，开工建设陇海路高架和农业路高架快速路，加快三环快速路、西三环北延、北三环东延、南三环东延等道路工程建设。加快城市路网建设，修通城市支线路网26条。充分利用地下空间，加快停车场、步行系统等静态交通的规划建设，新增公共停车泊位1万个以上。继续优先发展城市公共交通，加快中心城区公交换乘站、首末站、枢纽站建设，新购置公交车500辆，新开通公交线路20条，优化线路50条。提高公共设施保障能力。开工建设刘湾水厂、马头岗污水处理厂二期等市政工程，加大自来水、供热、供气等管网新建和改造力度，加快推进积水点改造、雨污水管网建设，主汛期前确保完成37处积水点改造任务。加大城中村改造力度，完善公共配套设施，提高承载能力。加强城市管理。集中力量整治交通秩序和市容市貌，开展占道经营、户外广告、报刊亭、城市养犬、建筑垃圾运输车等专项整治活动。加强市政工程的施工组织和管理，确保各项工程协调、有序、安全、快速推进。完善数字化城市管理系统，推进城市管理的标准化、精细化。大力开展爱国卫生运动，巩固国家卫生城市创建成果。

（八）深入推进改革，继续扩大开放。积极稳妥地推进国企改革发展，做好郑州百货大楼、百文集团、开普集团等企业的改制工作。鼓励、支持和引导非公有制经济发展，营造各种所有制经济依法平等使用生产要素、公平参与市场、同等受到法律保护的体制环境。加快医药卫生体制改革，巩固完善新农合制度，积极探索公立医院改革，支持非公立医疗卫生机构发展。着力推进公益性文化事业单位和艺术表演团体的改革，探索设立文化产业投资基金，推动出版集团上市工作。深化投融资体制改革，规范投融资平台建设，增强企业自身造血功能和国有资本运营能力。做好36家省定重点上市后备企业的培育工作，全年力争4家企业在境内外上市。深化农村体制改革。实施城乡建设用地增减挂钩，探索集体建设用地流转机制，建立城乡统一的交易市场。健全农村土地承包经营权流转机制，推进农用地和农村集体资产产权制度改革，稳步推进土地规模经营。推进集体林权制度、水利投融资体制、县级供销社体制改革。推进县（市）户籍制度改革，逐步降低入户门槛，加快城镇化进程。

　　继续实施开放带动和大招商战略，加强与国内外500强、跨国公司、大型央企和知名民营企业的战略合作，力争再引进一批大项目。落实“引进来、走出去”战略。巩固驻地招商成果，加大对长三角、珠三角、环渤海地区等重点区域的引资力度，积极承接产业转移。着力提升招商质量和层次，重点引进高新技术、新材料、新能源、生物医药、节能环保、新能源汽车和物联网等新兴产业。高度重视对外贸易工作。做好服务外包工作，积极申报全国服务外包基地示范城市。进一步扩大对外经济技术合作，培育外经龙头企业，提升国际竞争力。

　　（九）持续推进节能减排，切实加强环境保护。加强节能降耗工作，重点对冶金、水泥耐材、化工、煤炭、电力、造纸、纺织等高耗能行业实施综合改造。推广可再生能源建筑应用技术，推进绿色建筑示范项目。加快循环经济发展，实施20个循环经济重点项目，支持粉煤灰、尾矿废渣等废弃物资源化利用，推进废钢铁、电子产品等废旧资源的再生利用。加强生态环境建设，启动国家生态市创建工作。加快生态水系建设，完成东风渠上游、潮河上游、贾鲁河等河道治理工程，加快推进常（庄）西（流）湖生态公园建设。市区新增绿地500万平方米，新植乔木50万株。继续实施林业生态市建设工程，新造林19万亩，抚育改造中幼林10万亩。加强森林生态城的管护工作，切实发挥生态效益。加快黄河湿地自然保护区和黄河湿地公园建设。巩固和提升国家环保模范城创建成果，加大污染物化学需氧量、二氧化硫、氨氮、氮氧化物的减排力度，饮用水源地水质达标率达到98%以上，市区空气质量达标率达到85%以上。推进农村环境综合整治工作。

　　（十）着力保障和改善民生，确保社会大局和谐稳定。以办好省、市确定的“十大实事”为重点，大力加强民生工程和社会事业建设。实施更加积极的就业政策。全市新增城镇就业13万人，农村劳动力转移就业10万人。加强对高校毕业生、失业人员、农民工等重点群体的就业服务与技能培训，推动全民创业。加强社会保障工作。不断完善社会保险制度，着力提高社会保险统筹层次，继续扩大社会保险参保覆盖面。建立低收入群体和困难群众社会救助、保障标准与物价上涨挂钩联动制度，完善企业退休人员基本养老金、城乡居民低保标准正常调整机制，加强残疾人、孤儿、五保户等困难群体的保障体系和服务体系建设。推进教育优先发展。市区新建、改扩建中小学校25所、幼儿园97所，在全市农村中小学校实施现代远程教育“班班通”工程。强力推动郑州职业教育园区建设，支持郑州幼师升专工作。推动优质教育资源倍增工程。大力实施人才强市战略，统筹抓好高层次人才和高技能人才队伍建设。发展医疗卫生事业。实施优质医疗资源倍增工程，新建、改扩建市二院、市三院等6所医院，支持河南省医疗中心、郑大一附院郑东院区、洛阳正骨医院郑州医院等项目建设。新增片医负责制单位6个，使我市片医单位服务人口达到300万人。对城乡低保对象、低收入家庭重病患者、重度残疾人和农村五保对象实施大病救助，启动提高农村儿童重大疾病医疗保障水平试点工作。强化精神卫生健康服务。加快安居工程建设。以公共租赁住房和廉租住房建设为重点，编制保障性住房发展规划，加强保障性住房用地供给，扩大建设规模。开工建设经济适用房165万平方米、廉租住房17万平方米，筹措公共租赁住房65万平方米，改造棚户区58万平方米。推进文化、体育、人口和计划生育等社会事业发展。实施文化惠民工程，继续建设一批农村文化大院、社区文化中心和农家书屋，加快郑州图书馆新馆（市民文化中心）等重点工程建设。做好大遗址保护规划编制工作，支持少林功夫申报世界非物质文化遗产，加快商城遗址公园项目建设。深入开展“扫黄打非”专项斗争，加强网吧、音像、图书等文化市场的管理。继续做好人口和计划生育工作，深化人口和计划生育综合改革，稳定低生育水平，促进人的全面发展。加强妇女儿童保护工作。维护残疾人的合法权益。大力推动慈善事业发展。完善社会组织扶持政策，支持社会工作者队伍和志愿者队伍建设。开展全民健身运动，发展竞技体育。加强精神文明建设，弘扬郑州精神，营造积极向上、文明健康的精神文化氛围。维护社会和谐稳定。加强非煤矿山、化工、烟花爆竹、道路交通、燃气、消防等重点行业的安全治理，完善重大事故隐患防控体系，坚决遏制重特大事故的发生。严格落实煤矿安全生产责任制，严厉打击违法违规行为。继续强化食品药品安全监管，加强餐厨废弃物的回收管理。健全社会治安防控体系，积极开展法律服务和法律援助，努力调处化解社会矛盾纠纷，解决信访突出问题。加强应急管理，有效防范并妥善处置群体性事件和突发公共事件。严厉打击各类违法犯罪行为，努力维护社会大局稳定，为人民群众营造安全、祥和的社会环境。

　　继续做好国防动员、人民防空工作，加强全民国防教育和民兵预备役建设，深入开展“双拥”共建活动。巩固平等、团结、互助、和谐的社会主义民族关系，发挥宗教界人士和信教群众在促进经济社会发展中的积极作用。继续做好统计、审计、外事、侨务、对台事务、社科研究、史志、档案、地质、气象和地震监测等工作。

四、加强政府自身建设

　　做好今年各项工作，全面推进郑州都市区建设，要坚持“三具两基一抓手”，深入推进“两转两提”，扎实开展“创先争优”活动，切实提高政府行政效能和施政水平，努力建设依法治理、务实为民、清正廉洁、人民满意的政府。

　　（一）转变职能，提高政府执行力。继续推进政府职能转变，切实发挥经济调节、市场监管、社会管理和公共服务职能。正确履行经济调节职能，综合运用经济、法律和行政手段推动结构调整和发展方式转变；加强市场监管职能，强化对涉及人民群众生命财产安全领域的监管，特别是把涉及人民群众身体健康和生命安全的食品、药品等作为监管重点；更加注重社会管理职能，深入推进社会矛盾化解和社会管理创新；更加注重公共服务职能，加强公共基础设施建设，健全公共财政政策，完善公共服务体系，提高公共服务质量。

　　（二）依法行政，提高政府公信力。认真执行人大及其常委会的决议和决定，自觉接受人大及其常委会的法律监督、工作监督和政协的民主监督，认真办理人大代表议案、建议和政协委员提案。广泛听取各民主党派、工商联、无党派人士意见，支持工会、共青团、妇联等人民团体发挥桥梁纽带作用。规范行政执法，建立健全行政决策制度和行政执法责任追究制度，做到严格执法、公正执法、文明执法。完善政务公开制度，切实保障群众的知情权、参与权和表达权。落实廉政建设责任制，严格执行部门预算、政府采购等制度，从源头上预防腐败。加强对重点领域、关键部位、特殊岗位的监督管理，开展违法转包工程、违法建设、违法经营等专项治理活动。重视舆论监督和网络监督，确保人民群众的监督权利得到充分行使。

　　（三）转变作风，提高政府推动力。简化办事程序，精简会议文件，改进会风文风，提高工作效率。弘扬求真务实的作风，反对形式主义、官僚主义，反对弄虚作假，不搞劳民伤财的“形象工程”、“政绩工程”。弘扬艰苦奋斗的作风，带头厉行节约，勤俭办事，降低行政成本。弘扬调查研究之风，做到问政于民、问需于民、问计于民，使政府工作思路、工作部署、政策措施更加符合民意。健全公务员考核评价、激励约束机制，深入开展人民满意公务员创建活动。

　　（四）务实为民，提高政府凝聚力。牢固树立宗旨意识，坚持以人为本、执政为民，着力解决就业、就学、就医、住房等人民群众最直接、最关心的问题。把群众满意不满意、高兴不高兴、拥护不拥护、赞成不赞成作为评判各项工作的最终标准，坚决纠正损害群众利益的不正之风，真心实意为群众排忧解难，努力实现好、维护好、发展好最广大人民的根本利益和合法权益。

　　各位代表！

　　告别了成就辉煌的“十一五”，承载着全市人民的热切期盼，我们踏上了“十二五”的伟大征程。我们深知，前进的道路上会遇到曲折和坎坷；我们坚信，郑州的明天将会更加美好。让我们紧密团结在以胡锦涛同志为总书记的党中央周围，高举中国特色社会主义伟大旗帜，深入贯彻落实科学发展观，解放思想，开拓创新，抢抓机遇，加快发展，努力实现“十二五”的良好开局，为加快郑州都市区建设、推动中原经济区建设作出新的更大的贡献。
